# Lateral Dispersal and Foraging Behavior of Entomopathogenic Nematodes in the Absence and Presence of Mobile and Non-Mobile Hosts

**DOI:** 10.1371/journal.pone.0129887

**Published:** 2015-06-16

**Authors:** Harit K. Bal, Parwinder S. Grewal

**Affiliations:** Department of Entomology, The Ohio State University, OARDC, Wooster, Ohio, United States of America; Chinese Academy of Sciences, CHINA

## Abstract

Entomopathogenic nematodes have been classified into cruisers (active searchers) and ambushers (sit and wait foragers). However, little is known about their dispersal and foraging behavior at population level in soil. We studied lateral dispersal of the ambush foraging *Steinernema carpocapsae* (ALL strain) and cruise foraging *Heterorhabditis bacteriophora* (GPS11 strain) from infected host cadavers in microcosms (0.05 m^2^) containing Wooster silt-loam soil (Oxyaquic fragiudalf) and vegetation in the presence or absence of non-mobile and mobile hosts. Results showed that the presence of a non-mobile host (*Galleria mellonella* larva in a wire mesh cage) enhanced *H*. *bacteriophora* dispersal for up to 24 hr compared with no-host treatment, but had no impact on *S*. *carpocapsae* dispersal. In contrast, presence of a mobile host (*G*. *mellonella* larvae) increased dispersal of *S*. *carpocapsae* compared with no host treatment, but had no effect on *H*. *bacteriophora* dispersal. Also *H*. *bacteriophora* was better at infecting non-mobile than mobile hosts released into the microcosms and *S*. *carpocapsae* was better at infecting mobile than non-mobile hosts, thus affirming the established cruiser-ambusher theory. However, results also revealed that a large proportion of infective juveniles (IJs) of both species stayed near (≤ 3.8 cm) the source cadaver (88-96% *S*. *carpocapsae*; 67–79% *H*. *bacteriophora*), and the proportion of IJs reaching the farthest distance (11.4 cm) was significantly higher for *S*. *carpocapsae* (1.4%) than *H*. *bacteriophora* (0.4%) in the presence of mobile hosts. *S*. *carpocapsae* also had higher average population displacement than *H*. *bacteriophora* in the presence of both the non-mobile (5.07 vs. 3.6 cm/day) and mobile (8.06 vs. 5.3 cm/day) hosts. We conclude that the two species differ in their dispersal and foraging behavior at the population level and this behavior is affected by both the presence and absence of hosts and by their mobility.

## Introduction

Dispersal is a one-way movement of individuals of a population moving collectively or alone, that results in variation in local density and spatial distribution [1]. Dispersal is not only essential for individual fitness but it also has implications for population dynamics, population genetics and gene flow, and species distribution [2–4]. Environmental variability plays a major role in the dispersal of organisms, which reduces inbreeding and resource competition, resulting in distribution of populations at different spatial scales [5–7]. In the case of entomopathogenic nematodes (EPNs) in the families, Heterorhabditidae and Steinernematidae, the presence or absence of hosts and differences in host life history (e.g., mobile vs. sedentary) may contribute to the environmental/habitat heterogeneity, influencing dispersal and spatial distribution of the population, as observed in several predatory species [8,9].

EPNs must locate suitable hosts to complete their life cycle, but information on their host finding behavior in the soil is limited due to difficulties in studying the microscopic roundworms in the soil environment. Laboratory studies indicate a dichotomy in the host finding behavior of EPNs and have classified them as cruisers (active searchers) and ambushers (sit and wait foragers) [10,11]. Cruisers, such as *Heterorhabditis bacteriophora*, are characterized by active mobility [10,11], an ability to orientate to volatile long-range host cues [12,13], and an ability to find below ground sedentary hosts [13,14]. On the other hand, ambushers, such as *Steinernema carpocapsae*, have been shown to have low mobility [10,11], ability to nictate or tail standing [11, 15], and a lack of response to long-range volatile cues [12, 13, 16, 17]. Ambushers respond to short-range host volatile cues either after contact with the host cuticle [18] or during bouts of tail standing [19, 20]. These strategies in fact represent 2 extreme modes of the foraging continuum in which some species, such as *S*. *feltiae*, neither nictate like ambushers [11] nor respond to long-range host volatile cues in a manner similar to cruisers [13, 18]. These “intermediate foragers” [13] are often less effective than ambushers and cruisers at parasitizing hosts on either the soil surface or deep in the soil profile, respectively.

Since host cues such as CO2 and other host related odorants, have been shown to attract or repel EPNs causing directional movement in laboratory studies [12, 13, 17, 20–26], we compared dispersal of *H*. *bacteriophora* and *S*. *carpocapsae* in the presence or absence of hosts which differed in mobility (mobile vs non-mobile). While ambushers are likely to be more effective at finding mobile hosts, cruisers are likely more effective at finding sedentary below ground hosts [13, 18, 19, 27]. The foraging behavior of EPNs has also been found to be habitat specific [28] and their dispersal being influenced by the presence or absence of vegetation [29] and presence [30, 31, 32, 33] or absence of hosts [29, 34, 35]. It has been recently discovered that in the absence of hosts, a small proportion of the population of the ambusher, *S*. *carpocapsae* disperses long distances in the soil resulting in the same average daily dispersal at the population level as the cruiser, *H*. *bacteriophora* [29]. The authors referred to this proportion of the *S*. *carpocapsae* population as a “sprinting” population. The same authors in another study showed that the “sprinting” trait is heritable and could be genetically selected for increased proportion of sprinters although there were trade-offs in nictation ability and reproduction potential [36]. In this study, we compared the dispersal of *S*. *carpocapsae* and *H*. *bacteriophora* in the presence and absence of non-mobile and mobile hosts in microcosms containing soil and vegetation. We hypothesized that compared with the absence of hosts, the dispersal of *S*. *carpocapsae* will be enhanced in the presence of mobile hosts and that of *H*. *bacteriophora* will be enhanced in the presence of non-mobile hosts. We also hypothesized that population displacement (dispersal at the population level) will be greater in *S*. *carpocapsae* compared with *H*. *bacteriophora* in the presence of hosts due to its deployment of sprinters which disperse rapidly to find hosts.

Studies on dispersal and foraging behavior of nematodes at the population level in heterogeneous soil environments are rare. This study fills this void by simultaneous examination of the dispersal and foraging behavior at the population level in soil of two EPN species with contrasting foraging strategies in the absence and presence of mobile and non-mobile hosts. It concludes that the two species differ in their dispersal and foraging behavior at the population level and this behavior is affected by both the presence and absence of hosts and by their mobility.

## Materials and Methods

### Source of nematodes and soil

Frozen (in liquid nitrogen) stocks of *Heterorhabditis bacteriophora* GPS11 strain (a cruiser) and *Steinernema carpocapsae* ALL strain (an ambusher) were obtained from our laboratory collection and new cultures were raised by infecting final instar wax moth larvae, *Galleria mellonella*, obtained from Vanderhoest Canning Company, St. Mary’s, Ohio, following methods described by [37]. For all experiments, we used nematode infected *G*. *mellonella* cadavers as a source of nematodes, rather than aqueous suspensions, to mimic natural emergence. To prepare these cadavers, 20 separate 5 cm diameter Petri dishes were set up and each last instar *G*. *mellonella* was exposed to approximately 400 freshly produced IJs of either *H*. *bacteriophora* or *S*. *carpocapsae* at room temperature (22 C) for 3 days [29]. The nematode infected cadavers were then transferred to individual White traps [38] and observed once daily to check for the initiation of IJ emergence. Cadavers that had just begun to release the IJs within the past 24 hr were selected for use in all experiments to minimize variation due to initiation of emergence among replicates.

Wooster silt loam (Oxyaquic fragiudalf) topsoil was collected from a corn field at The Ohio State University, Wooster, Ohio. Particle size distribution of the soil, determined using methods described by [39,40], was 26.2% clay, 2.6% sand and 61.8% silt. The pH of the soil was 7.11 and organic matter content was 3.6%. The soil, autoclaved at 121 C and 103.42 kPa pressure for 10 hr, was stored at room temperature for at least 7 days before use to allow any toxic volatiles to escape. After estimating the saturation capacity of the autoclaved topsoil, its moisture level was adjusted to field capacity (i.e., 24% w/w; -106 kPa) by adding autoclaved tap water to optimize IJ movement.

### Preparation of experimental microcosms

**Fig 1:**
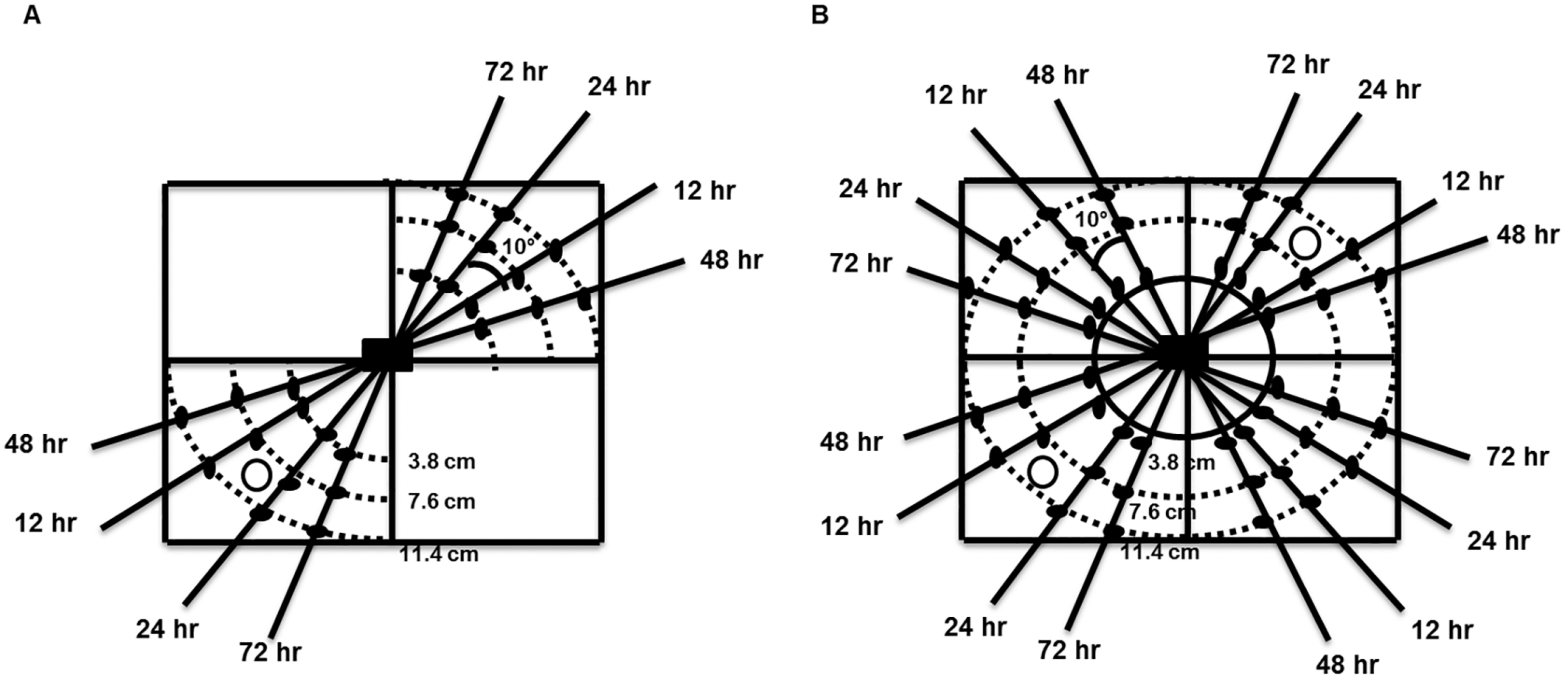
Experimental design. A pictorial representation of the experimental design showing 0.05 m^2^ sized microcosm used for studying dispersal of infective juveniles (IJs) emerging from the source nematode-infected cadaver (black rectangle) placed in the center in the presence of a non-mobile host (*Galleria mellonella* larva in a wire mesh cage) (**A**) and mobile hosts (*G*. *mellonella* larvae) (**B**). Each microcosm was divided into four quadrants. Host insects released in the microcosm are represented by empty circles. Soil cores samples were collected from 2 cm wide arcs (dotted lines) in the microcosms containing non-mobile host at different distances marked by black spots and at different time intervals depicted by the transects running through the microcosms at 12, 24, 48 and 72 hr, and then baited with live *G*. *mellonella* larvae in plastic cups (30 ml) to recover the dispersed IJs. Similarly, soil core samples were collected from 2 cm wide annuli (dotted circles) at different distances starting from the outer edge of the wire-mesh cylinder (bold circle) and at different time intervals in the microcosms with and without mobile hosts.

EPN dispersal was examined in 5 plastic microcosms (0.05 m2). Each microcosm was filled with autoclaved top soil to a depth of 5 cm and seeded with tall fescue (*Festuca arundinacea*) grass @ 34 g/m2. The microcosms were covered with white nylon fabric to allow for air exchange and light penetration for the grass to grow but to prevent any insect invasion and were held in the greenhouse at ~22 C during the course of each experiment. After the grass had grown to a height of approximately 2.5 cm, single 10-day old *G*. *mellonella* source cadaver that had just begun to release the IJs (see above) was placed 2.5 cm below the soil surface in the center of each microcosm to serve as the source of the IJs. In each microcosm, wooden sticks were inserted in the soil to mark specific distances from the cadaver in four transects separated by 10° angles (Fig 1).

### Lateral dispersal of EPNs in the presence or absence of non-mobile hosts

To study the influence of a non-mobile host on the lateral movement of the two nematode species, each microcosm was divided into 4 quadrants. One live *G*. *mellonella* larva enclosed in a wire mesh cage (1 x 1 x 1 cm; mesh size = 16) to restrict its movement (referred to as a non-mobile host) was buried 2.5 cm below the soil surface at 9 cm from the center in 1 of the quadrants (Fig 1A), at the same time when the 10-day old nematode infected *G*. *mellonella* cadaver was placed in the center of the microcosm (see above). One soil core sample (2 cm in diameter and 5 cm deep) was removed from each microcosm at 3.8, 7.6 and 11.4 cm from the center in the quadrant containing the non-mobile host and from the quadrant directly opposite at 12, 24, 48 and 72 hr after placing the source cadaver in the center and transferred to a plastic cup (30 ml). The holes left by the soil core samples were filled with the autoclaved soil to avoid any interference with subsequent nematode movement in the microcosms. Nematodes were recovered from the soil core samples using the insect baiting technique [37, 41], whereby each sample was baited with 1 uninfected last instar *G*. *mellonella* in each plastic cup, which was examined for nematode infection after 3 days, allowing sufficient time for nematodes of both species to infect the bait [42]. These cups were covered with lids containing 5 pin holes to allow for air exchange but to minimize moisture loss. Movement of at least one nematode from the cadaver to the site of the soil sample was inferred from the death of the baited insect showing characteristic symptoms of nematode infection. All infected larvae were dissected and the number of nematodes penetrating the bait insects was counted. Dissections were made on the third day after baiting to count the penetrated IJs before they had opportunity to reproduce. Five microcosms were used for each treatment and each experiment was performed twice resulting in n = 10 for each species at each distance and time point.

### Lateral dispersal of EPNs in the presence or absence of mobile hosts

The influence of a mobile host on the average lateral displacement and spatio-temporal patterns of the two species was also compared in the microcosms containing autoclaved field soil with vegetation. After preparing the microcosms as described above, a wire mesh cylinder (7.6 cm dia, 10 cm height; mesh size = 16) was inserted in the center of each microcosm (Fig 1B). At the time when the source cadaver was placed in the center of the microcosm, 2 last instar *G*. *mellonella* larvae were placed on the soil surface outside the wire mesh cylinder in 2 opposite quadrants, 1 in each (Fig 1B), to keep them away from the source cadaver. Since *G*. *mellonella* larvae could move anywhere in the microcosm in the area outside the wire mesh cylinder, nematode dispersal was tracked by collecting soil samples as described earlier at distances of 3.8, 7.6 and 11.4 cm starting at the external edge of the wire mesh cylinder outwards but in all 4 quadrants of the microcosm at 12, 24, 48 and 72 hr after burying the source cadaver (Fig 1B). Microcosms containing soil with vegetation but no *G*. *mellonella* larvae were set up as controls. Five microcosms were used for each treatment and each experiment was performed twice resulting in n = 10 for each species at each distance and time point.

### Data Analysis

The influence of host insects on the rate of movement and average displacement of the two species was determined by analyzing both the number of *G*. *mellonella* bait larvae killed and the number of IJs recovered from the baits in the microcosms. Mean percentage of IJs of both species dispersing at a time point up to a particular distance in a 2 cm wide arc in the microcosms in the presence and absence of non-mobile host was calculated by using the following equation.

$$D_{diti}\text{=}\left(M_{diti}/C_{di}\right)\times N{{}^{\circ}}_{di}\times f\times 100$$ (1)

where, M*_diti_* represents the mean number of IJs recovered from a soil core at a distance, *d* and a time point, *t*; C*_ti_* represents the mean cumulative number of IJs emerging from a 10-day old *G*. *mellonella* cadaver in the microcosm at a time point, *t* (obtained from [29]); N°*_di_* represents the total number of cores that would be extracted from an arc at a particular distance and *f* represents the correction factor used to account for differences in the penetration rates of the 2 species when exposed to *G*. *mellonella* larvae in soil [42]. Since the penetration rate of *S*. *carpocapsae* is 6 times greater than *H*. *bacteriophora* [42], we considered *f* = 6 for *H*. *bacteriophora* and *f* = 1 for *S*. *carpocapsae*.

$$\text{N}{{}^{\circ}}_{di}=\;\left(\text{Volume}\;\text{of}\;\text{an}\;\text{arc}\;\text{at}\;\text{distance},d\right)\;/\;\text{Volume}\;\text{of}\;\text{a}\;\text{soil}\;\text{core}$$

N°*_di_* = *d*, as radius of the soil core is 1 cm and depth is 5 cm.

In the presence and absence of mobile hosts, the mean percentage of IJs of both species dispersing at a time point up to a particular distance in a 2 cm wide annulus was calculated by eq 2 obtained by modifying eq 1.

$$D_{diti}\text{=}(M{{}^{\circ}}_{diti}\text{/}C_{ti})\times N_{di}\times f\times 100$$ (2)

where, M°*_diti_* represents the mean number of IJs recovered from 4 soil cores collected at a distance, *d* and a time point, *t* from all 4 quadrants in the microcosms and N*_di_* represents the total number of cores that would be extracted from an annulus at a particular distance (N*_di_* = 4*d*). All other parameters remain the same as described in eq 1.

While the mean proportion data obtained from the above two equations were transformed by the arcsine of the square root of the original proportions, the data on killed baits in the White traps and average displacement (cm/day) of both the species were transformed by the log10 (x+1), to achieve normality and equality of variance. Repeated measures analysis of variance (PROC GLM; Wilks’ lambda *F* statistic) was used to compare the mean number of infected *G*. *mellonella* baits and the mean proportion of IJs of each species over time between the presence and absence of mobile or non-mobile hosts. One-way analysis of variance (ANOVA) was used to test for significant difference in the average displacement (cm/day) of a species in the presence and absence of mobile or non-mobile hosts. Quadratic regression lines (PROC REG) were fitted between the mean number of infected *G*. *mellonella* baits, the mean proportion of IJs dispersed and the average displacement (cm/day) against time. Regression lines were fitted for each species in the presence as well as in the absence of both mobile and non-mobile hosts averaged over all distances and at individual distances from the source cadaver. The first derivative from each regression line was calculated and made equal to zero to find the maximum point. The linear and quadratic estimated coefficients obtained from the regression analyses were further used to compare the two species in the presence of mobile and non-mobile hosts and the 2 types of host within a species (PROC REG; TEST statement; SAS Release 9.3). Two-way tests were done between each species and each host type using both the linear and quadratic coefficients (*P* = 0.05) [43]. To compare the percentage of IJs between the 2 types of hosts for a species, the mean percentage of IJs expected to disperse to a particular distance in a 2 cm wide annulus was used for regression analysis. In the presence of non-mobile hosts, this was obtained by multiplying the mean percentage of IJs dispersing to a particular distance in a 2 cm wide arc calculated using eq 1 by 4. The data of all the repeated experiments were combined for all the analyses with repetition as a factor in ANOVA. There was no significant interaction between 2 trials and between trials and treatments for all the experiments (*P* > 0.05).

## Results

### Lateral dispersal of EPNs in the presence and absence of non-mobile hosts

Repeated measures analysis of variance did not show a significant change in the pattern of *H*. *bacteriophora* dispersal between the 3 distances, 3.8, 7.6 and 11.4 cm in the presence and absence of a non-mobile host both in the numbers of *G*. *mellonella* baits infected (F6,222 = 0.22; *P* = 0.97) and in the percentage of IJs (F6,222 = 0.90; *P* = 0.49) dispersed over a period of 12 to 72 hr after placing the source cadavers in the microcosms (Table 1). However, significantly higher mean number of infected *G*. *mellonella* baits as well as mean percentage of IJs was found in the presence of host than its absence at 12 hr (killed baits: F1,119 = 3.06; *P* = 0.05; IJs: F1,119 = 3.12; *P* = 0.04) and 24 hr (killed baits: F1,119 = 3.05; *P* = 0.05; IJs: F1,119 = 4.87; *P* = 0.03) after placing the source cadavers in the microcosms (Table 1). Although there was no significant interaction between distance and presence of the host in the number of infected baits (*P* ≥ 0.27) and percentage of IJs (*P* ≥ 0.20) at any time point, significantly greater numbers of baits were infected by *H*. *bacteriophora* at the closest arc, 3.8 cm from the source cadaver at 24 hr (F2,119 = 3.90; *P* = 0.02), 48 hr (F2,119 = 3.06; *P* = 0.05) and 72 hr (F2,119 = 5.94; *P* < 0.01), irrespective of the presence or absence of the host (Table 1). The average displacement of *H*. *bacteriophora* population, computed from all time intervals and distances did not differ in the presence and absence of the non-mobile host (Table 2). The non-mobile hosts placed in the microcosms were retrieved after 72 hr and dissected to confirm infection by *H*. *bacteriophora* IJs. Mean (± SE) number of IJs infecting each larva was found to be 52 ± 1.21.

**Table 1:** Lateral dispersal of *Heterorhabditis bacteriophora* in the presence and absence of non-mobile hosts.

Time after placing cadaver (hr)	Distance from cadaver (cm)	Host	No host	D	H	D*H	Host	No host	D	H	D*H
		Infected *G*. *mellonella* baits(Mean ± SE)					Percentage of infective juveniles(Mean ± SE)				
12	3.8	0.15 ± 0.07	0.00 ± 0.00 A	NS	**	NS	2.10 ± 0.69	0.00 ± 0.00 A	NS	**	NS
	7.6	0.15 ± 0.07	0.05 ± 0.05 A				2.01 ± 1.38	0.69 ± 0.69 A			
	11.4	0.00 ± 0.00	0.00 ± 0.00 A				0.00 ± 0.00	0.00 ± 0.00 A			
		a	b				a	b			
24	3.8	0.20 ± 0.09	0.05 ± 0.05 A	**	**	NS	0.56 ± 0.17	0.00 ± 0.00 A	NS	**	NS
	7.6	0.05 ± 0.05	0.00 ± 0.00 AB				0.18 ± 0.18	0.00 ± 0.00 A			
	11.4	0.00 ± 0.00	0.00 ± 0.00 B				0.00 ± 0.00	0.00 ± 0.00 A			
		a	b				a	b			
48	3.8	0.30 ± 0.10	0.35 ± 0.11 A	**	NS	NS	2.74 ± 2.34	0.26 ± 0.21 A	NS	NS	NS
	7.6	0.20 ± 0.09	0.25 ± 0.10 AB				0.35 ± 0.21	0.03 ± 0.03 A			
	11.4	0.10 ± 0.07	0.10 ± 0.07 B				0.11 ±0.07	0.00 ± 0.00 A			
		a	a				a	a			
72	3.8	0.50 ± 0.11	0.35 ± 0.11 A	**	NS	NS	0.08 ± 0.04	0.02 ± 0.01 A	NS	NS	NS
	7.6	0.25 ± 0.10	0.25 ± 0.10 AB				0.003 ± 0.003	0.01 ± 0.01 A			
	11.4	0.15 ± 0.08	0.05 ± 0.05 B				0.01 ± 0.01	0.004 ± 0.004 A			
		a	a				a	a			

Mean (± SE) number of infected *Galleria mellonella* baits from the collected soil core samples and mean (±SE) percentage of infective juveniles (IJs) of *Heterorhabditis bacteriophora* dispersed to a 2 cm wide arc at distances, 3.8, 7.6 and 11.4 cm over a period of 12 to 72 hr in the presence of a live non-mobile host, *G*. *mellonella* larva contained in a wire mesh cage buried in one of the quadrants in the microcosms containing autoclaved field soil with vegetation in comparison with the opposite quadrant of the same microcosm containing no host. Double asterisk (**) indicates significant difference between distances (D), presence or absence of the host (H) and interaction between the two (D*H) at a time point at *P* ≤ 0.05. Capital and small letters indicate Tukey’s comparison for means separation between distances averaged over presence or absence of the host, and presence and absence of host averaged over distances, respectively at a time point. NS = *P* > 0.05.

**Table 2:** Comparison of average population displacement of *Heterorhabditis bacteriophora* and *Steinernema carpocapsae* in the presence and absence of mobile and non-mobile hosts.

*Species*	*H*. *bacteriophora*			*S*. *carpocapsae*			Linear term	Quadratic term
	Presence and absence of non-mobile host							
Host	3.60 ± 0.12			5.07 ± 0.41			**	**
	(Y = 0.809–0.014 Time - 1.658 x 10^-4^ Time^2^, r^2^ = 0.02; *P* = 0.87)			(Y = 3.974–0.069 Time + 0.081 x 10^-2^ Time^2^, r^2^ = 0.08; *P* = 0.05)				
No host	3.37 ± 0.08			5.60 ± 0.45			**	**
	(Y = -0.184 + 0.027 Time - 2.564 x 10^-4^ Time^2^, r^2^ = 0.01; *P* = 0.15)			(Y = 5.611–0.101 Time + 4.387 x 10^-4^ Time^2^, r^2^ = 0.15; *P* < 0.01)				
	F	Df	*P*	F	df	*P*		
	2.37	1, 479	0.12	0.76	1, 479	0.38		
	Presence and absence of mobile host							
Host	5.30 ± 0.36			8.06 ± 0.59			**	**
	(Y = 13.217–0.351 Time + 0.003 Time^2^, r^2^ = 0.32; *P* < 0.01)			(Y = 4.737–0.097 Time + 6.558 x 10^-4^ Time^2^, r^2^ = 0.09; *P* = 0.02)				
No host	5.40 ± 0.25			4.20 ± 0.17			**	**
	(Y = 2.298 + 0.080 Time + 5.272 x 10^-4^ Time^2^, r^2^ = 0.02; *P* = 0.31)			(Y = 1.213–0.026 Time + 4.862 x 10^-4^ Time^2^, r^2^ = 0.04; *P* = 0.08)				
	F	Df	*P*	F	df	*P*		
	0.05	1, 479	0.81	52.41	1, 479	< 0.01		
Linear term	**			**				
Quadratic term	**			**				

Average (±SE) population displacement (cm/day) of infective juveniles (IJs) of *Heterorhabditis bacteriophora* and *Steinernema carpocapsae* dispersed to 2 cm wide annuli at all three distances, 3.8, 7.6 and 11.4 cm over a period of 12 to 72 hr in the presence and absence of mobile and non-mobile hosts in the microcosms containing autoclaved field soil with vegetation. Quadratic regression lines fitted for the average population displacement of IJs of each of the two species dispersed in the presence and absence of mobile and non-mobile hosts are bracketed. Double asterisk (**) indicates the significant difference in the estimated linear and quadratic coefficients between the two species, horizontally and the type of host (mobile *vs* non-mobile) within a species, vertically (from regression analyses, *P* ≤ 0.05). Analysis of variance indicates significant difference in the average population displacement of a species in the presence and absence of mobile and non-mobile hosts at *P* ≤ 0.05.

The presence of the non-mobile host did not significantly affect *S*. *carpocapsae* dispersal (Table 3). The mean number of infected *G*. *mellonella* baits (F6,222 = 0.99; *P* = 0.44) and the mean percentage of *S*. *carpocapsae* IJs (F6,222 = 0.74; *P* = 0.62) did not differ significantly between different distances over time (12 to 72 hr) in the presence and absence of a non-mobile host. There was no significant interaction between distance and the presence or absence of the host in the number of infected baits (*P* ≥ 0.19) and percentage of IJs (*P* ≥ 0.13) at any time point (Table 3). However, at the closest arc (3.8 cm) from the source cadaver, significantly higher mean numbers of infected baits were found at 12 hr (F2,119 = 7.91; *P* < 0.01), 24 hr (F2,119 = 10.99; *P* < 0.01) and 48 hr (F2,119 = 4.04; *P* = 0.03), and greater percentage of IJs were recovered at 12 hr (~88–98%; F2,119 = 3.27; *P* = 0.05) and 24 hr (94–98%; F2,119 = 6.58; *P* < 0.01) after placing the source cadavers, irrespective of the presence or absence of the host in the microcosm (Table 3). Overall, there was no significant difference in the average population displacement of *S*. *carpocapsae* IJs in the presence and absence of the non-mobile host (Table 2). The non-mobile host was infected by the nematodes and contained an average (± SE) of 139 ± 4.21 *S*. *carpocapsae* IJs per larva when dissected after the completion of the experiment at 72 hr.

**Table 3:** Lateral dispersal of *Steinernema carpocapsae* in the presence and absence of non-mobile hosts.

Time after placing cadaver (hr)	Distance from cadaver (cm)	Host	No host	D	H	D*H	Host	No host	D	H	D*H
		Infected *G*. *mellonella* baits(Mean ± SE)					Percentage of infective juveniles(Mean ± SE)				
12	3.8	0.50 ± 0.22	0.80 ± 0.17 A	**	NS	NS	38.20 ± 22.61	4.29 ± 2.05 A	**	NS	NS
	7.6	0.30 ± 0.21	0.30 ± 0.21 AB				0.86 ± 0.60	0.57 ± 0.36 B			
	11.4	0.00 ± 0.00	0.00 ± 0.00 B				0.00 ± 0.00	0.00 ± 0.00 B			
		a	a				a	a			
24	3.8	1.00 ± 0.00	1.00 ± 0.00 A	**	NS	NS	39.81 ± 25.90	40.02 ± 15.72 A	**	NS	NS
	7.6	0.80 ± 0.17	0.50 ± 0.22 A				2.00 ± 0.87	0.77 ± 0.43 B			
	11.4	0.15 ± 0.17	0.30 ± 0.21 B				0.49 ± 0.34	0.17 ± 0.17 B			
		a	a				a	a			
48	3.8	0.80 ± 0.17	0.30 ± 0.21 A	**	NS	NS	0.03 ± 0.01	0.06 ± 0.05 A	NS	NS	NS
	7.6	0.50 ± 0.22	0.30 ± 0.21 AB				0.05 ± 0.05	0.02 ± 0.01 A			
	11.4	0.15 ± 0.17	0.00 ± 0.00 B				0.02 ± 0.01	0.00 ± 0.00 A			
		a	a				a	a			
72	3.8	0.30 ± 0.21	0.30 ± 0.21 A	NS	NS	NS	0.02 ± 0.01	0.02 ± 0.01 A	NS	NS	NS
	7.6	0.30 ± 0.21	0.30 ± 0.21 A				0.02 ± 0.01	0.02 ± 0.01 A			
	11.4	0.30 ± 0.21	0.30 ± 0.21 A				0.01 ± 0.005	0.01 ± 0.005 A			
		a	a				a	a			

Mean (±SE) number of infected *Galleria mellonella* baits from the collected soil core samples and mean (±SE) percentage of infective juveniles (IJs) of *Steinernema carpocapsae* dispersed to a 2 cm wide arc at distances, 3.8, 7.6 and 11.4 cm over a period of 12 to 72 hr in the presence of a live non-mobile host, *G*. *mellonella* contained in a wire mesh cage buried in one of the quadrants in the microcosms containing autoclaved field soil with vegetation in comparison with the opposite quadrant of the same microcosm containing no host. Double asterisk (**) indicates significant difference between distances (D), presence or absence of the host (H) and interaction between the two (D*H) at a time point at *P* ≤ 0.05. Capital and small letters indicate Tukey’s comparison for means separation between distances averaged over presence or absence of the host, and presence and absence of host averaged over distances, respectively at a time point. NS = *P* > 0.05.

Quadratic regression lines were fitted for the mean number of *G*. *mellonella* baits infected by the two species and mean percentage of IJs of both species at all 3 distances from the source cadaver in the presence of a non-mobile host out of which, all the significant equations are presented in Table 4. When the estimated regression coefficients were compared between the 2 species, significantly greater number of baits were infected by *S*. *carpocapsae* than *H*. *bacteriophora* at all 3 arcs: 3.8 cm (Linear term: F1,77 = 14.08; *P* < 0.01; Quadratic term: F1,77 = 23.44; *P* < 0.01), 7.6 cm (Linear term: F1,77 = 5.37; *P* = 0.02; Quadratic term: F1,77 = 4.85; *P* = 0.03) and 11.4 cm (Linear term: F1,77 = 4.32; *P* = 0.04; Quadratic term: F1,77 = 3.97; *P* = 0.05). However, greater total percentage of *S*. *carpocapsae* (95.8%) than *H*. *bacteriophora* (67.3%) IJs were found at the closest arc, 3.8 cm (Linear term: F1,77 = 161.35; *P* < 0.01; Quadratic term: F1,77 = 55.49; *P* < 0.01) with no significant difference between the 2 (0.6% vs. 1.5%) at the farthest arc, 11.4 cm (*P* ≥ 0.29) from the source cadaver. In addition, significantly greater number of baits (Linear term: F1,237 = 8.64; *P* < 0.01; Quadratic term: F1,237 = 14.80; *P* < 0.01) were infected by *S*. *carpocapsae* and greater percentage of *S*. *carpocapsae* (Linear term: F1,237 = 111.40; *P* < 0.01; Quadratic term: F1,237 = 36.05; *P* < 0.01) than *H*. *bacteriophora* IJs dispersed up to the farthest annulus, 11.4 cm, in the presence of a non-mobile host over a period of 72 hr (Table 5). The average population displacement of *S*. *carpocapsae* was also significantly greater than *H*. *bacteriophora* in the presence of a non-mobile host (Table 2; Linear term: F1,237 = 3.87; *P* = 0.05; Quadratic term: F1,237 = 3.90; *P* = 0.05).

**Table 4:** Quadratic regression lines fitted for *Heterorhabditis bacteriophora* and *Steinernema carpocapsae* in the presence of hosts.

Host insect	Distance from cadaver (cm)	Species	Regression equation	r^2^	*P*
			Infected *G*. *mellonella* baits		
Non-mobile	3.8	Hb	Y = 0.063 + 0.004 Time + 2.879 x 10^-5^ Time^2^	0.12	0.01
		Sc	Y = 0.114 + 0.045 Time - 5.932 x 10^-4^ Time^2^	0.27	0.03
	7.6	Hb	Y = 0.055 + 0.001 Time + 2.094 x 10^-5^ Time^2^	0.04	0.20
		Sc	Y = 0.170 + 0.022 Time - 2.137 x 10^-4^ Time^2^	0.03	0.75
	11.4	Hb	Y = -0.042 + 0.002 Time + 2.181 x 10^-6^ Time^2^	0.07	0.06
		Sc	Y = 0.147–0.010 Time + 1.439 x 10^-4^ Time^2^	0.10	0.31
Mobile	3.8	Hb	Y = 0.477 + 0.016 Time - 2.582 x 10^-4^ Time^2^	0.18	0.07
		Sc	Y = 1.001–0.001 Time - 8.755 x 10^-5^ Time^2^	0.55	< 0.01
	7.6	Hb	Y = -0.244 + 0.025 Time - 2.505 x 10^-4^ Time^2^	0.21	0.05
		Sc	Y = 0.392 + 0.007 Time - 7.415 x 10^-5^ Time^2^	0.01	0.87
			Percentage of infective juveniles		
Non-mobile	3.8	Hb	Y = -0.012 + 0.001 Time - 0.192 x 10^-4^ Time^2^	0.02	0.51
		Sc	Y = 0.622–0.016 Time + 1.036 x 10^-4^ Time^2^	0.17	0.13
Mobile	3.8	Hb	Y = 0.763–0.029 Time + 2.669 x 10^-4^ Time^2^	0.65	< 0.01
		Sc	Y = 1.681–0.059 Time + 4.995 x 10^-4^ Time^2^	0.58	< 0.01
Mobile	11.4	Hb	Y = -0.003 + 2.302 x 10^-4^ Time - 2.502 x 10^-6^ Time^2^	0.14	0.14
		Sc	G = 0.020–6.364 x 10^-4^ Time + 5.309 x 10^-6^ Time^2^	0.17	0.09

Quadratic regression lines fitted for the mean number of *Galleria mellonella* baits infected by *Heterorhabditis bacteriophora* (Hb) and *Steinernema carpocapsae* (Sc) and mean percentage of IJs of the two species at a particular distance from the source cadaver in a microcosm over a period of 12 to 72 hr in the presence of non-mobile and mobile hosts.

**Table 5:** Comparison of lateral dispersal of *Heterorhabditis bacteriophora* and *Steinernema carpocapsae* in the presence of mobile and non-mobile hosts.

*Species*	*H*. *bacteriophora*	*S*. *carpocapsae*	Linear term	Quadratic term
Host insect	Dead *G*. *mellonella* baits (Mean ± SE)			
Non-mobile	0.16 ± 0.02	0.40 ± 0.06	**	**
	(Y = 0.025 + 0.002 Time + 0.173 x 10^-4^ Time^2^; r^2^ = 0.06; *P* < 0.01)	(Y = 0.144 + 0.018 Time - 0.221 x 10^-3^ Time^2^; r^2^ = 0.03; *P* = 0.36)		
Mobile	0.29 ± 0.04	0.49 ± 0.04	NS	NS
	(Y = 0.062 + 0.015 Time - 1.764 x 10^-4^ Time^2^; r^2^ = 0.03; *P* = 0.24)	(Y = 0.464 + 0.005 Time - 8.132 x 10^-5^ Time^2^; r^2^ = 0.02; *P* = 0.39)		
Linear term	**	NS		
Quadratic term	**	NS		
Host insect	Percentage of infective juveniles (Mean ± SE)			
Non-mobile	2.71 ± 0.94	26.62 ± 12.57	**	**
	(Y = 0.021 + 6.318 x 10^-4^ Time - 1.176 x 10^-5^ Time^2^; r^2^ = 0.04; *P* = 0.61)	(Y = 0.848–0.022 Time + 0.141 x 10^-3^ Time^2^; r^2^ = 0.05; *P* = 0.15)		
Mobile	6.64 ± 1.75	14.92 ± 4.21	**	**
	(Y = 0.293–0.011 Time + 9.517 x 10^-5^ Time^2^; r^2^ = 0.21; *P* < 0.01)	(Y = 0.631–0.022 Time + 1.891 x 10^-4^ Time^2^; r^2^ = 0.20; *P* < 0.01)		
Linear term	**	NS		
Quadratic term	**	NS		

Mean (±SE) number of dead *Galleria mellonella* baits from the collected soil core samples and mean (±SE) percentage of infective juveniles (IJs) of *Heterorhabditis bacteriophora* and *Steinernema carpocapsae* dispersed to 2 cm wide annuli at all three distances, 3.8, 7.6 and 11.4 cm over a period of 12 to 72 hr in the presence of mobile and non-mobile host in the microcosms containing autoclaved field soil with vegetation. Quadratic regression lines fitted for the mean number of dead *G*. *mellonella* baits and mean percentage of IJs of each of the two species dispersed up to 11.4 cm distance from the source cadaver over a period of 72 hr in the presence of mobile and non-mobile hosts are bracketed. Double asterisk (**) indicates the significant difference in the estimated linear and quadratic coefficients between the two species, horizontally and the type of host within a species, vertically (from regression analyses, *P* ≤ 0.05). NS = *P* > 0.05.

*Steinernema carpocapsae* also showed significantly greater average displacement than *H*. *bacteriophora* in the absence of the host (Table 2; Linear term: F1,237 = 41.63; *P* < 0.01; Quadratic term: F1,237 = 8.96; *P* < 0.01). This is evident from significantly higher mean number of *G*. *mellonella* baits (Linear term: F1,237 = 3.67; *P* = 0.05; Quadratic term: F1,237 = 3.84; *P* = 0.05) infected by *S*. *carpocapsae* (Y = 0.501 + 0.001 Time - 1.786 x 10^-4^ Time^2^, r^2^ = 0.03; *P* = 0.33) than *H*. *bacteriophora* (Y = -0.126 + 0.010 Time - 0.727 x 10^-4^ Time^2^, r^2^ = 0.09; *P* < 0.01) as well as greater mean percentage of *S*. *carpocapsae* (Y = 0.029 + 0.003 Time - 4.613 x 10^-5^ Time^2^, r^2^ = 0.04; *P* = 0.19) than *H*. *bacteriophora* (Y = 0.004–0.015 x 10^-2^ Time - 0.136 x 10^-5^ Time^2^, r^2^ = 0.01; *P* = 0.52) IJs dispersed in the quadrant containing no host over a period of 72 hr (Linear term: F1,237 = 217.71; *P* < 0.01; Quadratic term: F1,237 = 445.36; *P* < 0.01).

### Lateral dispersal of EPNs in the presence and absence of mobile hosts

Although repeated measures analysis of variance did not show a significant change in the pattern of *H*. *bacteriophora* dispersal between the 3 distances in the presence and absence of mobile hosts in the number of *G*. *mellonella* baits infected (F6,222 = 1.41; *P* = 0.22) over time (12 to 72 hr), the mean percentage of IJs (F6,222 = 2.21; *P* = 0.05) varied significantly between different distances over time (Table 6). Specifically, significantly higher mean number of infected *G*. *mellonella* baits was found in the absence of host than its presence at 24 hr (F1,119 = 10.13; *P* < 0.01), 48 hr (F1,119 = 7.98; *P* = 0.01) and 72 hr (F1,119 = 36.12; *P* < 0.01) after placing the source cadavers in the microcosms and at the closest annulus, 3.8 cm from the cadaver at 12 hr (Distance*Host, F2,119 = 3.95; *P* = 0.02) (Table 6). Additionally, a significantly greater mean percentage of *H*. *bacteriophora* IJs dispersed from 3.8 to 11.4 cm in the presence of host at 12 hr (F1,119 = 4.93; *P* = 0.03) and in its absence at 24 hr (F1,119 = 4.93; *P* = 0.03), 48 hr (F1,119 = 6.88; *P* = 0.01) and 72 hr (F1,119 = 10.79; *P* < 0.01) and at the shortest distance, 3.8 cm at 24 hr (Distance*Host, F2,119 = 3.31; *P* = 0.04) (Table 6). Irrespective of the presence or absence of the mobile hosts, significantly greater numbers of killed baits were found at the closest arc, 3.8 cm from the source cadaver at all the time points, 12 hr (F2,119 = 100.44; *P* < 0.01), 24 hr (F2,119 = 22.28; *P* < 0.01), 48 hr (F2,119 = 13.14; *P* < 0.01) and 72 hr (F2,119 = 3.84; *P* = 0.03), and significantly greater percentages of IJs were recovered at 12 hr (F2,119 = 47.26; *P* < 0.01) and 24 hr (F2,119 = 4.44; *P* = 0.02) (Table 6). *Heterorhabditis bacteriophora* did not differ in the average displacement in soil with and without mobile hosts (Table 2). When dissected, each mobile host larva released in the microcosm was found to be infected with 22 ± 0.52 IJs, 72 hr after placing the source cadavers.

**Table 6:** Lateral dispersal of *Heterorhabditis bacteriophora* in the presence and absence of mobile hosts.

Time after placing cadaver (hr)	Distance from cadaver (cm)	Host	No host	D	H	D*H	Host	No host	D	H	D*H
		Infected *G*. *mellonella* baits(Mean ± SE)					Percentage of infective juveniles(Mean ± SE)				
12	3.8	0.64 ± 0.11	0.90 ± 0.10 A	**	NS	**	49.36 ± 9.87	31.59 ± 5.13 A	**	**	NS
	7.6	0.07 ± 0.04	0.00 ± 0.00 B				8.70 ± 5.54	0.00 ± 0.00 B			
	11.4	0.00 ± 0.00	0.00 ± 0.00 B				0.00 ± 0.00	0.00 ± 0.00 B			
		a	a				a	b			
24	3.8	0.71 ± 0.16	0.90 ± 0.10 A	**	**	NS	12.00 ± 3.11	100.00 ± 51.51 A	**	**	**
	7.6	0.14 ± 0.11	0.70 ± 0.15 B				5.80 ± 2.08	8.91 ± 8.91 B			
	11.4	0.00 ± 0.00	0.10 ± 0.10 C				0.00 ± 0.00	8.91 ± 8.91 B			
		b	a				b	a			
48	3.8	0.68 ± 0.13	0.90 ± 0.10 A	**	**	NS	1.77 ± 0.38	12.15 ± 4.71 A	NS	**	NS
	7.6	0.46 ± 0.16	0.80 ± 0.13 A				1.50 ± 0.51	5.10 ± 1.24 A			
	11.4	0.07 ± 0.04	0.30 ± 0.15 B				0.30 ±0.17	2.54 ± 1.69 A			
		b	a				b	a			
72	3.8	0.32 ± 0.09	1.00 ± 0.00 A	**	**	NS	0.06 ± 0.01	7.98 ± 2.82 A	NS	**	NS
	7.6	0.28 ± 0.15	0.80 ± 0.13 AB				0.11 ± 0.06	4.56 ± 1.95 A			
	11.4	0.07 ± 0.04	0.60 ± 0.16 B				0.04 ± 0.02	0.60 ± 0.17 A			
		b	a				b	a			

Mean (±SE) number of infected *Galleria mellonella* baits from the collected soil core samples and mean (±SE) percentage of infective juveniles (IJs) of *Heterorhabditis bacteriophora* dispersed to a 2 cm wide annulus at distances, 3.8, 7.6 and 11.4 cm over a period of 12 to 72 hr in the presence of 2 mobile hosts, *G*. *mellonella* larvae placed outside the wire mesh cylinder (3.8 cm dia) enclosing the source cadaver in the microcosms containing autoclaved field soil with vegetation as opposed to similar microcosms containing no hosts. Double asterisk (**) indicates significant difference between distances (D), presence or absence of the host (H) and interaction between the two (D*H) at a time point at *P* ≤ 0.05. Capital and small letters indicate Tukey’s comparison for means separation between distances averaged over presence or absence of the host, and presence and absence of host averaged over distances, respectively at a time point. NS = *P* > 0.05.

The influence of mobile hosts on *S*. *carpocapsae* dispersal is evident from the significant change in the mean number of infected *G*. *mellonella* baits (F6,222 = 3.12; *P* = 0.01) and mean percentage of IJs (F6,222 = 7.57; *P* < 0.01) between different distances over time, 12 to 72 hr (Table 7). While significantly higher mean number of killed baits as well as mean percentage of IJs in the killed baits was found in the presence of hosts than their absence at 12 hr (Killed baits: F1,119 = 29.83; *P* < 0.01; IJs: F1,119 = 34.45; *P* < 0.01) and 24 hr (Killed baits: F1,119 = 9.42; *P* < 0.01; IJs: F1,119 = 4.64; *P* = 0.03) after placing the source cadavers in the microcosms, these values were significantly lower with hosts than without at 72 hr (Killed baits: F1,119 = 13.22; *P* < 0.01; IJs: F1,119 = 7.16; *P* = 0.01) (Table 7). The closest annulus, 3.8 cm from the source cadaver contained significantly higher numbers of killed baits at 12 hr (Distance*Host, F2,119 = 3.75; *P* = 0.03) and 48 hr (Distance*Host, F2,119 = 5.83; *P* < 0.01) and significantly greater percentage of IJs at 12 hr (Distance*Host, F2,119 = 24.40; *P* < 0.01), 24 hr (Distance*Host, F2,119 = 3.26; *P* = 0.04) and 48 hr (Distance*Host, F2,119 = 4.10; *P* = 0.02) in the presence of mobile hosts (Table 7). In addition, irrespective of the presence or absence of the mobile hosts, significantly higher mean number of infected baits as well as mean percentage of IJs was found at the shortest distance, 3.8 cm at 12 hr (Killed baits: F2,119 = 19.92; *P* < 0.01; IJs: F2,119 = 27.39; *P* < 0.01), 24 hr (Killed baits: F2,119 = 21.87; *P* < 0.01; IJs: F2,119 = 12.94; *P* < 0.01) and 48 hr (Killed baits: F2,119 = 25.66; *P* < 0.01; IJs: F2,119 = 4.84; *P* = 0.01) (Table 7). The mobile hosts were found to be infected with an average (± SE) of 735 ± 8.24 *S*. *carpocapsae* IJs per larva, 72 hr after placing the source cadavers. Overall, the average displacement of *S*. *carpocapsae* was significantly greater in microcosms containing mobile hosts compared with no hosts (Table 2).

**Table 7:** Lateral dispersal of *Steinernema carpocapsae* in the presence and absence of mobile hosts.

Time after placing cadaver (hr)	Distance from cadaver (cm)	Host	No host	D	H	D*H	Host	No host	D	H	D*H
		Infected *G*. *mellonella* baits(Mean ± SE)					Percentage of infective juveniles(Mean ± SE)				
12	3.8	0.93 ± 0.04	0.30 ± 0.15 A	**	**	**	100.00 ± 23.20	3.09 ± 2.05 A	**	**	**
	7.6	0.43 ± 0.10	0.00 ± 0.00 B				12.73 ± 5.04	0.00 ± 0.00 B			
	11.4	0.14 ± 0.07	0.00 ± 0.00 B				1.47 ± 0.72	0.00 ± 0.00 B			
		a	b				a	b			
24	3.8	1.00 ± 0.00	0.60 ± 0.16 A	**	**	NS	51.98 ± 18.72	16.80 ± 8.13 A	**	**	**
	7.6	0.61 ± 0.04	0.20 ± 0.13 B				3.96 ± 1.37	0.53 ± 0.35 B			
	11.4	0.11 ± 0.07	0.00 ± 0.00 C				0.42 ± 0.29	0.00 ± 0.00 B			
		a	b				a	b			
48	3.8	0.68 ± 0.07	0.90 ± 0.10 A	**	NS	**	0.91 ± 0.37	6.85 ± 2.63 A	**	NS	**
	7.6	0.50 ± 0.07	0.20 ± 0.13 B				0.89 ± 0.39	0.06 ± 0.04 B			
	11.4	0.32 ± 0.07	0.00 ± 0.05 B				0.37 ± 0.17	0.00 ± 0.00 B			
		a	a				a	a			
72	3.8	0.46 ± 0.11	0.90 ± 0.10 A	NS	**	NS	0.23 ± 0.09	4.66 ± 1.96 A	NS	**	NS
	7.6	0.50 ± 0.13	0.80 ± 0.13 A				0.19 ± 0.08	7.13 ± 3.17 A			
	11.4	0.25 ± 0.14	0.70 ± 0.15 A				0.08 ± 0.05	1.25 ± 0.53 A			
		b	a				b	a			

Mean (±SE) number of infected *Galleria mellonella* baits from the collected soil core samples and mean (±SE) percentage of infective juveniles (IJs) of *Steinernema carpocapsae* dispersed to a 2 cm wide annulus at distances, 3.8, 7.6 and 11.4 cm over a period of 12 to 72 hr in the presence of 2 mobile hosts, *G*. *mellonella* larvae placed outside the wire mesh cylinder (3.8 cm dia) enclosing the source cadaver in the microcosms containing autoclaved field soil with vegetation as opposed to similar microcosms containing no hosts. Double asterisk (**) indicates significant difference between distances (D), presence or absence of the host (H) and interaction between the two (D*H) at a time point at *P* ≤ 0.05. Capital and small letters indicate Tukey’s comparison for means separation between distances averaged over presence or absence of the host, and presence and absence of host averaged over distances, respectively at a time point. NS = *P* > 0.05.

Of all the quadratic regression lines fitted for the mean number of killed baits and mean percentage of IJs of the 2 species dispersed to different distances from the source cadaver in the presence of mobile hosts, all significant ones are presented in Table 4. Comparison of estimated regression coefficients showed that significantly greater percentage of *S*. *carpocapsae* (3.8 cm: 88.4%; 11.4 cm: 1.4%) than *H*. *bacteriophora* (3.8 cm: 79.3%; 11.4 cm: 0.4%) IJs dispersed at the closest annulus, 3.8 cm (Linear term: F1,77 = 21.32; *P* < 0.01; Quadratic term: F1,77 = 9.70; *P* < 0.01) and the farthest annulus, 11.4 cm (Linear term: F1,77 = 55.74; *P* < 0.01; Quadratic term: F1,77 = 33.46; *P* < 0.01) from the source cadaver. There was no significant difference between the 2 species in the mean number of infected baits at any distance from the source cadaver (*P* ≥ 0.09). Overall, significantly greater percentage of *S*. *carpocapsae* (Linear term: F1,237 = 10.15; *P* < 0.01; Quadratic term: F1,237 = 5.02; *P* = 0.03) than *H*. *bacteriophora* IJs dispersed up to the farthest annulus, 11.4 cm from the source cadaver in the presence of mobile hosts over a period of 72 hr (Table 5). In addition, *S*. *carpocapsae* showed significantly higher average displacement than *H*. *bacteriophora* in the presence of mobile hosts (Table 2; Linear term: F1,237 = 10.13; *P* < 0.01; Quadratic term: F1,237 = 4.84; *P* = 0.03).

In the absence of mobile hosts, *H*. *bacteriophora* showed significantly greater average displacement than *S*. *carpocapsae* (Table 2; Linear term: F1,237 = 7.26; *P* = 0.01; Quadratic term: F1,237 = 4.88; *P* = 0.03). Significantly higher mean number of baits (Linear term: F1,237 = 3.41; *P* = 0.05; Quadratic term: F1,237 = 4.92; *P* = 0.03) was infected by *H*. *bacteriophora* (Y = 0.141 + 0.018 Time - 0.123 x 10^-3^ Time^2^, r^2^ = 0.13; *P* < 0.01) than *S*. *carpocapsae* (Y = 0.121–6.979 x 10^-5^ Time + 1.291 x 10^-4^ Time^2^, r^2^ = 0.27; *P* < 0.01); however, the 2 species did not differ significantly (*P* ≥ 0.31) in the mean percentage of IJs dispersed over a period of 72 hr in the absence of mobile hosts.

### Lateral dispersal of EPNs in the presence of mobile vs non-mobile hosts

In case of *H*. *bacteriophora*, comparison of estimated regression coefficients between the 2 types of hosts showed greater mean number of infected *G*. *mellonella* baits (Linear term: F1,237 = 5.48; *P* = 0.02; Quadratic term: F1,237 = 9.78; *P* < 0.01) and mean percentage (Linear term: F1,237 = 27.83; *P* < 0.01; Quadratic term: F1,237 = 17.87; *P* < 0.01) of IJs that dispersed up to 11.4 cm annulus over a period of 72 hr in the presence of mobile than non-mobile hosts (Table 5). Specifically, significantly higher numbers of killed baits were found at annuli, 3.8 cm (Linear term: F1,77 = 3.92; *P* = 0.05; Quadratic term: F1,77 = 4.99; *P* = 0.03) and 7.6 cm (Linear term: F1,77 = 3.92; *P* = 0.05; Quadratic term: F1,77 = 4.99; *P* = 0.03) from the source cadaver and greater percentage (Linear term: F1,77 = 40.19; *P* < 0.01; Quadratic term: F1,77 = 27.14; *P* < 0.01) of *H*. *bacteriophora* IJs remained within the closest annulus, ≤ 3.8 cm in the presence of mobile (79.3%) than non-mobile (67.3%) hosts (see Table 4 for regression equations). *Heterorhabditis bacteriophora* also showed significantly greater average displacement in the presence of mobile than non-mobile (Table 2; Linear term: F1,237 = 8.73; *P* < 0.01; Quadratic term: F1,237 = 4.12; *P* = 0.04) hosts.

Although *S*. *carpocapsae* showed no significant differences either in the mean number of infected *G*. *mellonella* baits (*P* ≥ 0.14) or in the mean percentage of IJs in the killed baits (*P* ≥ 0.63) in the presence of mobile and non-mobile hosts (Table 5), it showed significantly higher average displacement in the presence of mobile than non-mobile hosts (Table 2; Linear term: F1,237 = 6.57; *P* = 0.01; Quadratic term: F1,237 = 3.81; *P* = 0.05). There was no significant difference in the number of killed baits (*P* ≥ 0.09) or percentage of dispersed IJs (*P* ≥ 0.07) at any distance from the source cadaver in the presence of mobile compared with non-mobile hosts.

## Discussion

The results of this study show that the presence of both a non-mobile and mobile host can result in small but significant increases in dispersal of *H*. *bacteriophora* in soil. However, the influence of the host on dispersal of *H*. *bacteriophora* was transient as it lasted only for the first 12 hr in case of the mobile host and 24 hr in case of the non-mobile host. Also the presence of neither the non-mobile nor mobile host had any effect on the average population displacement of *H*. *bacteriophora*. The rapid early increase in the dispersal of *H*. *bacteriophora* in the presence of hosts indicates a quick orientation of the IJs towards the uninfected hosts [12, 13]. The subsequent death of the hosts due to nematode infection closely mimics the fate of the hosts in the natural conditions. The rapid detection of the hosts may have resulted in fewer *H*. *bacteriophora* IJs being recovered from the soil in the microcosms containing mobile hosts than the ones with no hosts, perhaps due to the IJs infecting the found hosts. The lack of persistent effect of hosts on *H*. *bacteriophora* dispersal beyond the initial 12–24 hr period may also be due to the reduction in attraction of the host to the IJs arriving after it had already been infected by sufficient number of the pioneering IJs. Reduction in the attractiveness of infected hosts to conspecific IJs has been previously demonstrated by [17] who reported that infected hosts become progressively less attractive or even repellent after a brief period of increased early attractiveness to conspecific IJs (i.e., recruitment). This lack of attraction or even repellence of the infected hosts may have even resulted in the IJs switching to a ranging search, thus dispersing into other quadrants in the microcosm to find more suitable hosts.

The presence of a mobile host had a significant and substantial positive impact on the dispersal of *S*. *carpocapsae*, but a non-mobile host had little effect. The positive effect of the mobile host on *S*. *carpocapsae* dispersal was particularly strong during the first 12 hr and it lasted for up to 24 hr. This strong effect of the mobile host suggests that *S*. *carpocapsae* sprinters are able to respond rapidly to chemical or other cues from mobile hosts just like cruisers [12, 20, 22, 26, 44] to increase their directed movement towards the source of the cue(s). Although there is evidence of EPN attraction to indirect cues such as herbivore induced plant volatiles [45–50], we intentionally used a host that feeds on bees wax and does not feed on plant parts. Earlier studies have shown CO2 emissions to cause EPN orientation and aggregations [12, 20, 22, 25]. CO2 has also been recently reported to be a critical host-seeking cue for EPNs regardless of their host-seeking strategy and also an essential cue for attraction to *G*. *mellonella* [26]. Further, the absence of CO2 leads to reduction in chemotaxis in both *H*. *bacteriophora* and *S*. *carpocapsae* [26]. Therefore, greater dispersal of *S*. *carpocapase* in response to mobile than non-mobile hosts may be explained by likely higher amounts CO2 emitted as a by-product of respiration by actively moving hosts. Greater EPN attraction to the vibrations created by host movement in the soil [51] could also be a possible reason of greater average displacement of both EPN species in the presence of mobile hosts. Insects are known to transmit acoustic stimuli in soil up to 20 cm [52], but response of EPNs to such stimuli is unknown. The lack of continuous positive impact of hosts on *S*. *carpocapsae* dispersal beyond the initial 24 hr period may be due to reduced attractiveness or even repellency of the infected hosts to conspecific IJs as explained by [17].

Interestingly, greater proportion of *S*. *carpocapsae* IJs were recovered at the farthest distance (11.4 cm) from the source cadaver at all the time points in the microcosms containing mobile hosts as opposed to the ones with no hosts where few sprinting IJs were found only after 72 hr of placing the source cadavers. The detection of sprinting IJs at the farthest distance as quickly as 12 hr in the presence of mobile hosts suggests that *S*. *carpocapsae* sprinters may be able to use chemical or other cues from the mobile host to increase their nictation or jumping behavior [19, 53] to move faster than in the absence of hosts. Campbell and Gaugler [11] reported a 100-fold reduction in host finding by *S*. *carpocapsae* as opposed to 19-fold by *H*. *bacteriophora* when movement of the potential host *Tenebrio molitor* was restricted. We have found *S*. *carpocapsae* sprinters to retain some nictation ability even under negative selection pressure [36]. Therefore, we speculate that enhanced nictation or jumping by the dispersing sprinters in the presence of mobile than non-mobile hosts could likely further contribute to the enhanced dispersal of *S*. *carpocapsae*.

Dissection of the hosts released into the microcosms revealed that *H*. *bacteriophora* was better at infecting non-mobile than mobile hosts, and *S*. *carpocapsae* was better at infecting mobile than non-mobile hosts. These results affirm that cruisers are better at finding sedentary hosts and ambushers are better at finding mobile hosts [13, 18, 19, 27, 54]. Furthermore, this study supports the hypothesis that *S*. *carpocapsae* population displacement (dispersal at the population level) is greater than *H*. *bacteriophora* in the presence of hosts. This is likely due to the deployment of a small number of sprinting IJs by *S*. *carpocapsae* that quickly disperse from the natal cadaver to find hosts. There was a significantly greater increase in the dispersal of *S*. *carpocapase* compared with *H*. *bacteriophora* in the presence of hosts, irrespective of host mobility. This is also evident from significantly greater average population displacement of *S*. *carpocapsae* than *H*. *bacteriophora* in the presence of both the non-mobile (5.07 vs. 3.6 cm/day) and mobile (8.06 vs. 5.3 cm/day) hosts. Although significantly higher percentage of *S*. *carpocapsae* than *H*. *bacteriophora* IJs stayed near the immediate vicinity of the source cadaver at 3.8 cm distance in the presence of both mobile and non-mobile hosts, significantly greater numbers of infected baits as well as percentage of IJs of *S*. *carpocapsae* than *H*. *bacteriophora* were recovered at the farthest distance, 11.4 cm, in the presence of non-mobile and mobile hosts. In the absence of hosts and vegetation, *S*. *carpocapsae* and *H*. *bacteriophora* have been reported to exhibit an aggregated group movement behavior in sand-filled arenas 3 days after cadaver application [55] and similar average population displacement in soil-filled microcosms even up to 10 days [29]. In the presence of both mobile and non-mobile hosts, there was a considerable population of *H*. *bacteriophora* (67–79%) that remained within the close vicinity of the source cadaver, although it was less than that of *S*. *caprpocasae* (88–96%). Waiting close to the natal cadaver may be an adaptive strategy to conserve energy to enhance survival [56] whereby few individuals may rapidly respond to host cues while the others may remain immobile and disperse only after they receive information about the suitability of the host from kin [17]. Differential infectiousness among individuals of *H*. *bacteriophora* population [57–59] may be another explanation for a proportion of *H*. *bacteriophora* population staying close to the natal cadaver even in the presence of hosts. Overall, our results on the nematode dispersal patterns reveal dichotomy in the dispersal behavior of both species.

Evolutionary ecology theory suggests that there must always be some offspring that leave the natal habitat to found new populations [60–62]. For EPNs dependent on ephemeral resources, one might expect all offspring to be dispersing at least some distance. The results of this study demonstrate that a majority of the population in both species stays near the source cadaver (67–79% for *H*. *bacteriophora* and 88–96% for *S*. *carpocapsae*), at least during the first 72 hr, while a small percentage of the population (0.6–1.4% for *S*. *carpocapsae* and 0.4–1.5% for *H*. *bacteriophora*) disperses much further away (11.4 cm) from the source host cadaver in the presence of hosts. As *S*. *carpocapsae* reproduce sexually (as opposed to *H*. *bacteriophora* which are hermaphroditic), they must not only find a host but also IJs of both sexes must invade the same host. With so few IJs dispersing from the cadaver over much longer distances than the rest of the population, it is difficult to see how this is achieved. One possibility is the recruitment of the slow-moving followers into the hosts infected by the fast-moving sprinters at farther distances from the source cadavers. Grewal et al. [23] put forth the hypothesis that male acts as a colonizing sex and showed that in four of the five *Steinernema* species, the IJs destined to become males not only dispersed faster and farther than those destined to become females they also invaded hosts first making them more suitable and attractive for the following female IJs to find and invade them, thus enhancing the reproductive success of the entire population. Other studies have also shown evidence of kin recruitment into the recently infected hosts [17, 24], emergence of male *S*. *carpocapsae* IJs before the female IJs from the host cadavers [63], and sex-related communication between adult nematodes stimulated by chemical cues or pheromones [64–67]. Further, using the same microcosms, Bal et al. [36] found that *S*. *carpocapsae* responds positively to selection for enhanced dispersal via increasing the proportion of sprinters and by shifting towards a male-biased sex ratio. Therefore, the results of this study and the above discussion suggest that EPNs, particularly *S*. *carpocapsae*, may use a foraging strategy akin to “scouting” in which a small number of individuals disperse rapidly, scanning a large area to find suitable hosts while the rest of the population stays near the natal cadaver likely anticipating information about potential hosts, thus conserving energy and reproductive success of the entire population. We anticipate further research on the scouting theory.
